# Microbial Protein for Human Consumption: Towards Sustainable Protein Production

**DOI:** 10.1111/nbu.70028

**Published:** 2025-09-17

**Authors:** Anthony W. Watson, Rebecca F. Townsend, Matt Longshaw

**Affiliations:** ^1^ School of Biomedical, Nutritional and Sports Sciences Newcastle University Newcastle upon Tyne UK; ^2^ Human Nutrition and Exercise Nutrition Centre Newcastle University Newcastle upon Tyne UK; ^3^ Calysta UK Ltd Newcastle upon Tyne UK

**Keywords:** consumer acceptance, digestibility, fermentation, microbial protein, sustainability

## Abstract

Protein from animal sources significantly contributes to greenhouse gas emissions, driving the need for sustainable alternative protein sources to meet global dietary demands while reducing environmental impact. This project explores microbial protein, derived through cellular agriculture using fermentation technology, as a viable, sustainable and high‐quality protein for human consumption. This report describes a multidisciplinary approach to assessing the feasibility of incorporating microbial protein into human food systems, guided by four key objectives. First, a market analysis to identify opportunities and challenges for incorporating microbial protein into existing food products, assessing its potential to improve the protein quality of plant‐based foods. Second, the project will evaluate the protein quality and digestibility of reformulated products using advanced models simulating human gastrointestinal processes. Third, consumer perceptions and barriers to adopting bacterial‐based proteins will be investigated, addressing safety, health and sustainability concerns. Overall findings will inform the development of a technical document outlining actionable recommendations for commercialising microbial proteins as food ingredients. This multidisciplinary project aims to support the sustainable diversification of dietary protein sources, contributing to global efforts towards achieving sustainable food systems. The project is funded by the Start Healthy, Stay Healthy (STAR) Hub, a Diet and Health Open Innovation Research Club (OIRC) which is funded by the UK Research and Innovation (UKRI) Biotechnology and Biological Sciences Research Council (BBSRC).

## 1 Introduction

Protein derived from animal products is a major factor in greenhouse gas emissions (GHG) with an estimated 57% of GHG from food production coming from animal‐based agriculture (Xu et al. 2021). There is also an acknowledgement that the intensification of commodity production, such as soybean, in recent decades has resulted in significant socioeconomic and environmental consequences including intensified land use, deforestation and interconnected (peri‐coupled) socioeconomic impacts (da Silva et al. 2021). The pursuit of alternative proteins is one of the key areas of focus of the Food and Agriculture Organization's (FAO) Programme Priority Area on Bioeconomy for Sustainable Food and Agriculture which aims to support the achievement of Sustainable Development Goal target 12.2—Sustainable management and efficient use of natural resources. There is a recognition that if we are to sustainably provide sufficient protein for the global population, we need to diversify available sources of dietary protein and become less reliant on animal‐derived protein.

These climate factors along with animal welfare concerns are nudging consumers to source alternative protein food options in a way that has driven the UK market for non‐animal derived alternatives to a value of £942 million in 2023 (GFI‐Europe 2024).

This drive towards a shift from ‘conventional animal’ protein sources to alternative sources has created space for products such as plant‐based meat and milk alternatives (Smith et al. 2024). Interestingly, hybrid products which contain both animal and plant‐based proteins in an attempt to improve the sustainability of traditional animal‐based products are also becoming available for the flexitarian market. In general, plant‐based animal alternatives are of lower protein quality than their animal counterparts. This is generally attributed to an incomplete amino acid profile, with plant‐based protein generally being lower in essential amino acids such as methionine and lysine (Young and Pellett 1994) and/or reduced digestibility and net protein utilisation due to interactions with other components within the foods such as fibre and polyphenolic compounds such as tannins (Cirkovic Velickovic and Stanic‐Vucinic 2018; Sarwar Gilani et al. 2012). It must however be acknowledged that considerable research and development has been undertaken in the area of plant‐based meat alternatives to combine different plant sources of protein with complementary EAA profiles to improve levels of limiting amino acids and improve the protein chemical score of the foods. However, the appearance of EAAs in human plasma after ingestion of such foods is still generally lower than that of their meat alternatives (Pham et al. 2022).

Emerging technologies and consumer demand have created an environment for new novel alternative proteins to be conceptualised, researched and in some cases commercialised. The most successful of these is mycoprotein, which was developed and commercialised by Quorn in the United Kingdom in the 1980s. This research area has developed considerably with the emergence of lab‐grown muscle protein and cellular agriculture such as bacteria produced through fermentation.

**FIGURE 1:**
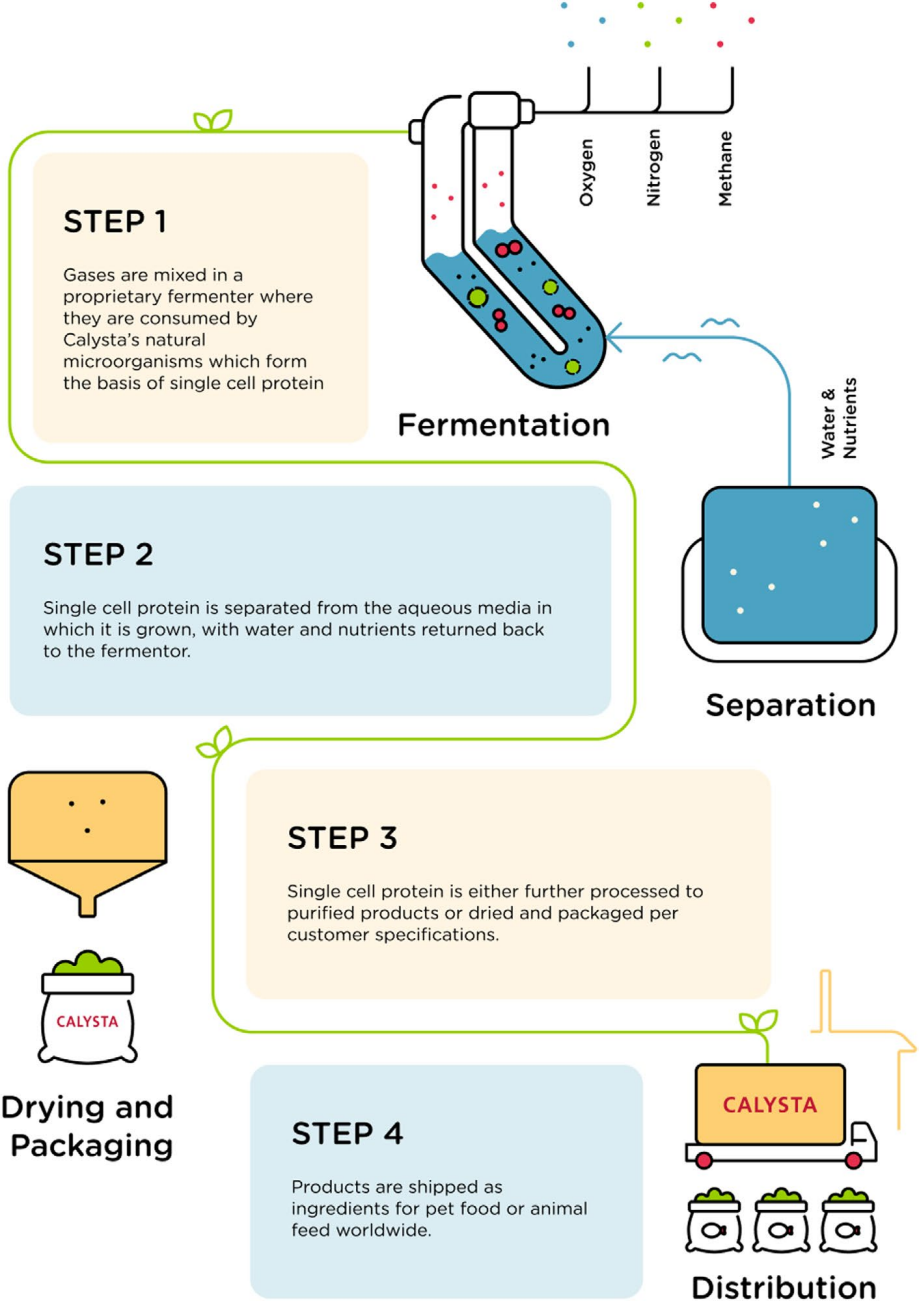
Microbial protein production.

Cellular agriculture utilises fermentation technology to grow bacteria such as *Methylococcus capsulatus* that convert carbon and energy into high‐quality, non‐GMO protein sources. The product is produced in bioreactors, which allow production at scale using very little water and agricultural land compared to conventional agriculture (Pikaar et al. 2018), to create sustainable, traceable and affordable protein (see Figure 1). Importantly, the microbes convert nitrogen into cellular proteins with an efficiency close to 100% (Pikaar et al. 2017) with the resulting microbial proteins constituting greater than 70% of the dry biomass weight (Oba et al. 2023) producing an estimated 4.6 kg of CO_2_ per kg of final product. In comparison, in the United Kingdom, the CO_2_ equivalent per kg of product produced is conservatively estimated at 17.12 kg for beef and 14.6 kg for sheep (AHDB 2010) and the global median for soybean production is estimated to be 2 kg (Poore and Nemecek 2018).

The amino acid profile of the microbial protein is excellent, with all essential amino acids present in good quantities and a digestibility of > 85% in animal models (Oba et al. 2023). The high quality of the microbial protein gives potential for use in mixed protein source products and could be used to improve the protein quality of food formulations such as plant‐based meat alternatives and ready to eat foods and milks, which have overall poor protein quantity and quality, where one or more essential amino acids are limiting.

The project described here aims to explore the feasibility of using bacterial protein as a food ingredient and inform the next steps needed to commercialise the protein product in the human food market.

The project is funded by the Start Healthy, Stay Healthy (STAR) Hub, a Diet and Health Open Innovation Research Club (OIRC) innovation hub which is funded by the UK Research and Innovation (UKRI) Biotechnology and Biological Sciences Research Council (BBSRC). ORIC promotes collaborations between industry and academia in order to foster interdisciplinary partnerships to accelerate research translation, support sustainable food systems and enable healthier lifestyles across all stages of life (https://oirc.org.uk/). The project will run for 15 months (August 2024–November 2025) and has four key objectives related to the development and dissemination of guidelines for the formulation of palatable, cost‐effective, higher‐protein foods which incorporate microbial protein produced by Calysta UK Ltd.

## 2 Objective One: Conduct a Market Assessment to Describe Current Trends in Non‐Animal‐Based Protein Products, With the Goal of Informing the Development of Reformulated Foods That Incorporate Microbial Proteins

The UK plant‐based food market was estimated to be worth £942 million in 2024. The market decreased overall by 2.8% between 2022 and 2023 but there are key segments of the market that are still in growth, including cheese, cream and milks (GFI‐Europe 2024). There are analogues made using soybean that have good amino acid profiles (Kudełka et al. 2021) and a Digestible Indispensable Amino Acid Score (DIAAS) of 90 (van den Berg et al. 2022); however, many of the products now available, such as market‐leading milk and yoghurt alternatives, are produced using grains such as oats (Yu et al. 2023) almonds, or rice (Pucci et al. 2024) which have a lower quality amino acid profile when compared to their animal‐derived counterparts. This provides an opportunity to create hybrid products with bacterial protein added to create non‐animal‐derived products with good protein quantity and quality. This objective comprises two components:

### 2.1 Market Analysis

Market analysis will focus on identifying the opportunities, challenges and strategies for including microbial protein within existing products. Using the most up to date market data, key segments of interest will be identified and key products within those segments will be chosen for development. Literature will be reviewed to allow us to choose those foods which would most benefit from the inclusion of microbial protein. This will include considerations of key product categories and consumer segments as well as potential for protein quality improvements.

### 2.2 Product Development

Foods selected through the market analysis will undergo reformulation using total protein and protein chemical score calculations as guiding metrics. Desk‐based calculations based on essential amino acid profiles of the chosen products from published literature will be included. Chemical scores will be estimated based on published amino acid profiles before and after the addition of the microbial protein based on the recommendations by WHO 2013. The products will be formulated based on UK food regulations of ‘a source of protein’ (protein as 12% or more of total energy) and ‘high in protein’ (protein as 20% or more of total energy). Products will be prepared, including cooking where necessary, and assessed in a Digestible Indispensable Amino Acid Score (DIAAS) like gut model.

## 3 Objective Two—Assessment of Protein Quality and Digestibility

The gold standard for the measurement of quality and digestibility of protein in foods is through DIAAS. DIAAS, which measures the digestibility of protein at the end of the small intestine, requires a growing pig to be fed and harvested for the assessment to be conducted. There is a growing call within the scientific community for models to be produced to allow DIAAS to be assessed through laboratory‐based methods instead of animals. Aelius Biotech Ltd. has developed a proprietary model of the human gastrointestinal tract that enables the study of various products, including foods, functional foods, nutraceuticals, formulations and pharmaceuticals, and their interactions within the digestive system. This advanced model integrates digestion and absorption in a single system using a patented mucus layer. This innovation transforms any 2D cell culture system into a 3D model that accurately simulates digestion and absorption (Chater et al. 2023).

The Human Gastrointestinal Model provides detailed insights into how products behave in the digestive system. The model is particularly useful for assessing protein quality, offering measurements like DIAAS. This score evaluates protein quality based on its true ileal digestibility. The system not only calculates the DIAAS and provides a detailed amino acid profile but also tracks digestion rates over time throughout the digestive tract.

This work package will utilise the Aelius biotech model to understand the quality and digestibility of the proteins in products created in objective one.

## 4 Objective Three: Understand the Consumer Perceptions and Barriers to Consuming Bacteria‐Based Proteins

Recent consumer perceptions report into alternative proteins by the European Institute of Innovation and Technology (2023) found that there are three key consumer segments with differing perspectives on protein. These are ‘meat lovers’, ‘plant purists’ and ‘omnivores’. It was reported that meat lovers and plant purists hold positive opinions about their protein food segments and the group which has the greatest potential for alternative proteins to be positioned are omnivores. However, some misconceptions need to be clarified around safety, benefits to health and environmental sustainability. Similar findings were reported by the UK Food Standards Agency, where safety concerns were identified as a key barrier to consuming insect‐based or lab‐grown proteins. In regard to encouraging uptake, 42% of respondents reported that nothing could encourage them to try lab‐grown meat, but 27% could be persuaded if they knew it was safe to eat, and 23% if they trusted that it was properly regulated (Jarchlo Ibrahim 2022). These findings from Europe and the UK highlight that some of the consumer perceptions of alternative proteins involve major concerns around safety and healthiness of insects or lab‐grown proteins. To our knowledge, there are no reports which aim to gauge consumer perceptions of fermented bacterial‐based proteins. The overall aim of this work package is to explore consumer perceptions surrounding microbial protein, to gauge the likelihood of future inclusion into their diet.

This work package will involve an explanatory sequential mixed methods design. The quantitative phase will involve conducting a cross‐sectional survey among a UK‐representative sample to understand the behavioural factors influencing consumer acceptance of incorporating bacterial protein into a diet. This will also include how use of semantics may influence uptake. Relevant frameworks, such as The Theoretical Domains Framework (TDF) (Atkins et al. 2017) and the Theoretical Framework of Acceptability (TFA) (Sekhon et al. 2017) will be used to guide the development of survey items. Survey data will be analysed using appropriate statistical methods and will inform the qualitative phase of the work package, through the development of a semi‐structured topic guide for use in a series of focus groups. The aim of this phase is to examine the behavioural influences in further depth, alongside prospective acceptability of the products developed within WP 1 to 2. This phase aims to recruit a diverse sample of participants to aid generalisability of results to the wider UK population. Template analysis will then be used to aid interpretation of focus group data, involving the development of a coding template with inductive and deductive themes.

## 5 Objective Four: Collate Project Findings to Produce a Technical Document

Building on outcomes one, two and three, academic and industry stakeholders will create a document that outlines the efficacy and feasibility of producing food‐safe versions of Calista's proteins. The document will integrate experimental results, market analysis and consumer insights to outline the potential of microbial proteins as a sustainable and high‐quality food ingredient. It will also provide actionable recommendations for the commercialisation and regulatory approval of these proteins.

## 6 Conclusion

The Microbial Protein for Human Consumption project will use a multidisciplinary approach to provide an evidence base for the use of microbial protein in human food systems. The project will provide valuable information on the efficacy of reformulating products using microbial protein to improve the protein quality of commonly consumed foods and the barriers and opportunities for their commercialisation.

## Author Contributions

Anthony W. Watson, Rebecca F. Townsend and Matt Longshaw all contributed equally to the writing and preparation of the manuscript. Academic team: Dr. Anthony W. Watson, Newcastle University (Principal Investigator); Dr. Rebecca F. Townsend, Newcastle University (Co‐Investigator); Dr. Matt Longshaw, Calysta UK (Co‐Investigator).

## Conflicts of Interest

Matt Longshaw is an employee of Calysta UK Ltd. who has made an in‐kind contribution to the project. Anthony W. Watson has in the past received funding from the food industry and has received financial compensation for non‐food industry facing consultancy. Rebecca F. Townsend declares no conflicts of interest.

## Data Availability

The data that support the findings of this study are available from the corresponding author upon reasonable request.
